# Separation of the Symphysis Pubes

**Published:** 1854-07

**Authors:** D. L. M’Gugin


### *Separation of the Symphysis Pubes.*

BY PROF. D. L. M'GUGIN.

Mrs. W. pregnant with ninth child and was delivered in Nov. 1851, of a living and healthy fœtus weighing about 9 pounds. When taken in labor the pains were regular but feeble, and as her previous labors were somewhat protracted, immediate assistance was not secured.— There was soon, however, a rapid increase of the uterine effort, and immediately the membranes were ruptured and the liquor amnii discharged. There was an inclination to evacuate the bowels, a delusion often occurring, and the result of the pressure of the fœtal head upon the lower rectum. For this purpose she left her bed and while over the chamber a strong expulsive uterine effort came on when she was assured the child would soon be expelled and efforts were made by her to protect the child from injury in a fall upon the floor. For this purpose the hands were extended back to the vulva while her body was in a curved position in which situation, by a second powerful expulsive effort, the child was born. Until this moment she was alone and unassisted, but aid now being at hand she was conveyed to bed. Soon, however, she was convinced that something unusual had taken place, as it was with great difficulty she could move her lower limbs, the attempt to do so occasioning great suffering, referable to the pubic region. The next day in the evening advice was solicited, when she was found to be suffering much with fever, pain in the head, hot skin, pulse 110, pain in the hypogastric region, which was increased by deep pressure, the urine had not been evacuated, pain upon the slightest movement of the muscles of the lower extremities, and cough occasioning the most extravagant suffering at the symphysis, the viscera of the abdomen being driven down upon it. If one limb was extended while the other was being flexed by assistants, a kind of low rubbing sound was heard and a perceptible movement felt at the symphysis. This was also produced by pressing alternately upon the spinous processes of the ossa ilia. By placing the fingers of one hand upon the anterior facet of one descending ramus of the pubes, and those of the other hand in like manner upon the other, then a movement of the limbs would produce a perceptible movement of the pubic bones upon one another at the symphyseal junction. The cracking sound observed by some authors in these accidents, was not heard in this, but there was the strongest evidence that the articulation had given way. The result was that an inflammation occurred in the bladder, the uterus, the ovaries and the peritoneal reflexion over these organs, demanding the antiphlogistic treatment which was energetically employed. The urine was drawn off by the catheter which was required to be used as often as the urine collected, a roller was applied to prevent muscular movement by the limbs or the muscles of the pelvis, opiates to procure rest and allay the harrassing cough, which treatment was followed by her recovery, slowly but steadily.

The size of the child, the extraordinary expulsive force, and the position of the woman were circumstances which would favor such an accident, and it is hardly to be supposed it would have occurred had it not been that there was probably then existing a relaxation of the symphyseal ligaments which often occurs in the latter months of utero gestation, and in those who have borne many children. Dr. Meigs gives an interesting case at page 38 of his "Treatise on Obstetrics" to which the reader is referred. Prof. Hughes also saw this case, concurred in opinion, and aided in its management.

I have not learned that the subject of the case I have related has been since pregnant.—*Trans. Iowa State Med. Society.*

